# Comparison of the Number and Reasons for Self-Perceived Barriers to Accessing Primary Health Care Services Between Roma and Ethnic Albanians

**DOI:** 10.2478/sjph-2026-0002

**Published:** 2026-03-01

**Authors:** Alvi Naum, Albana Gjyzari, Gentiana Qirjako, Katarzyna Czabanowska, Ervin Toçi, Genc Burazeri

**Affiliations:** Department of International Health, CAPHRI (Care and Public Health Research Institute), Maastricht University, Maastricht, The Netherlands; Local Health Care Unit, Korça, Albania; University of Medicine, Rr. “Dibres”, No. 371, Tirana, Albania; Institute of Public Health, Tirana, Albania; Institute of Public Health, Faculty of Health Sciences, Jagiellonian University, Krakow, Poland

**Keywords:** Access to health services, Albania, Barriers, Ethnic differences, Primary health care, Roma and Egyptian minorities, dostop do zdravstvenih storitev, Albanija, ovire, etične razlike, primarne zdravstvene storitve, manjšine Romov in Egipčanov

## Abstract

**Introduction:**

To compare the number and reasons for self-perceived barriers to accessing primary health care (PHC) services between Roma/Egyptian and ethnic Albanians.

**Methods:**

533 adults (mean age: 45±18 years; ≈60% women) reporting barriers to accessing PHC services were recruited consecutively during a nationwide survey in October 2024 across all four regions of Albania, using probability-proportional-to-size sampling. A semi-structured questionnaire was administered by trained interviewers inquiring about the number and reasons for self-perceived barriers to accessing PHC services, health characteristics, and sociodemographic factors. General linear models and binary logistic regression were employed to assess the association between perceived barriers and ethnic groups.

**Results:**

444 (≈83%) participants were ethnic Albanians, whereas the remaining 89 (≈17%) individuals belonged to other ethnic groups, including Roma (n = 57), Egyptians (n = 30), and Gorani or Macedonians (n = 2). Overall, cost and waiting time were the most common barriers. Roma/Egyptian minorities faced more cultural and language issues, whereas Albanians reported higher distrust and service-related expectations. The crude mean number of barriers to accessing PHC services was higher among Roma/Egyptian minorities than among Albanians (1.8 vs. 1.6, respectively; P = 0.04). The multivariable-adjusted odds of reporting ≥ 2 barriers to accessing PHC services were 93% higher among Roma/Egyptian minorities than in Albanians (P = 0.03).

**Conclusions:**

Roma/Egyptian minorities experience more barriers in accessing PHC services than ethnic Albanians. However, the cost of services constitutes the main barrier across both groups. Conversely, communication-related barriers affect mainly Roma/Egyptian minorities, whereas Albanians perceive more systemic barriers. In Albania, there is a need for targeted, equity-focused interventions.

## Izvleček

**Uvod:**

Primerjava števila in razlogov za samopercipirane ovire pri dostopu do primarnih zdravstvenih storitev (PZS) med Romi/Egipčani in etničnimi Albanci.

**Metode:**

V okviru nacionalne raziskave smo oktobra 2024 zaporedno rekrutirali 533 odraslih (povprečna starost: 45 ± 18 let; ≈ 60 % žensk), ki so poročali o ovirah pri dostopu do PZS, v vseh štirih regijah Albanije z uporabo vzorčenja sorazmernega verjetnostnega vzorca glede na velikost. Izurjeni anketarji so izvedli polstrukturirani vprašalnik o številu in razlogih za samopercipirane ovire pri dostopu do PZS, zdravstvenih značilnostih in sociodemografskih dejavnikih. Za oceno povezave med zaznanimi ovirami in etničnimi skupinami smo uporabili splošne linearne modele in binarno logistično regresijo.

**Rezultati:**

Večina, ali 444 (≈ 83 %), udeležencev je bilo etničnih Albancev, preostalih 89 (≈ 17%) pa je pripadalo drugim etničnim skupinam, vključno z Romi (n = 57), Egipčani (n = 30) ter Gorani ali Makedonci (n = 2). Skupno so bile najpogostejše ovire stroški in čas čakanja. Manjšine Romov/Egipčanov so se soočale z več kulturnimi in jezikovnimi težavami, medtem ko so Albanci poročali o večjem nezaupanju in pričakovanjih v zvezi s storitvami. Groba povprečna številka ovir pri dostopu do PZS je bila višja pri manjšinah Romov/Egipčanov v primerjavi z Albanci (1,8 proti 1,6; P = 0,04). Večvariabilno prilagojene verjetnosti za poročanje o ≥ 2 ovirah pri dostopu do PZS so bile med Romi/Egipčani za 93 % višje kot med Albanci (P = 0,03).

**Zaključki:**

Manjšine Romov/Egipčanov se pri dostopu do PZS soočajo z več ovirami kot etnični Albanci. Vendar stroški storitev predstavljajo glavno oviro v obeh skupinah. Nasprotno pa ovire v povezavi s komunikacijo prizadenejo predvsem Rome/Egipčane, medtem ko Albanci zaznavajo več sistemskih ovir. V Albaniji so potrebne ciljno usmerjene intervencije, osredotočene na enakopravnost.

## 1 INTRODUCTION

Across numerous European countries, Roma communities face significant barriers to healthcare access related to poverty, discrimination, language barriers and geographic isolation, which collectively contribute to widespread unmet health needs (1,2,3,4,5). This is a matter of particular concern, as Roma are often recognised as the largest ethnic minority group in Europe (2, 6). According to the World Health Organization (WHO), between ten and twelve million Roma reside in the WHO European Region, with approximately six million living in the European Union (EU) alone (6).

The designation of “Roma” as a singular ethnonym does not adequately reflect the complex and heterogeneous reality of the various communities it encompasses, each characterised by distinct ethnic, historical, and cultural identities (1, 7). In its official documentation, the EU employs the umbrella term “Roma” to collectively denote multiple distinct groups (8).

Terminology aside, overall, the Roma minorities experience a substantial amount of discrimination throughout Europe (1,2,3,4, 9, 10), which is one of the important reasons for unmet healthcare needs in these ethnic groups (1,2,3,4,5, 9, 10). Nevertheless, in countries of Central and Eastern Europe, including Albania, the main reason for unmet health needs among Roma minorities consists of the affordability of care (4). Besides service costs, other key structural barriers to accessing healthcare include geographic distance, physical accessibility, and transportation difficulties (11). In addition to these structural constraints, cultural and language mismatches represent communication-related barriers that further hinder access to healthcare services (12). Beyond these factors, interpersonal or experiential barriers, such as lack of trust or dissatisfaction when expectations are not met, also deter service use and may be shaped by deeper systemic issues within the healthcare system (13).

A comprehensive study including twelve Central and Eastern European countries has documented a higher proportion of Roma individuals with unmet health needs compared with their non-Roma counterparts (4). The prevalence of unmet health needs among Roma participants varied from ≈13% in Montenegro to ≈66% in Moldova, with Albania (≈60%) exhibiting the second highest estimate (4). Furthermore, the magnitude of the positive association between Roma status and unmet health needs was highest in Albania among the other Central and Eastern European countries (4).

In Albania, there are two ethnic groups under the “Roma” denomination, namely Roma and Egyptian minorities (14). According to the last census conducted in Albania in April 2023 (14), the Roma and Egyptian minorities (N = 22,188 inhabitants) comprise about 1% of the Albanian population. However, it is widely acknowledged that census data significantly underrepresents marginalised and hard-to-reach populations such as Roma minorities. A considerable segment of this population maintains a highly mobile lifestyle. Besides the remarkably high prevalence of unmet health needs (4), the available evidence pertaining to Roma minorities in Albania suggests several important barriers to accessing healthcare services, especially primary health care (PHC) services, such as discrimination, lack of identification documents, language and cultural barriers, as well as low health literacy levels (15). Furthermore, a recent report identified important gaps in service quality for Roma and Egyptian minorities in Albania, including discrimination, provider attitudes, and administrative barriers (16).

Nevertheless, the available information about Roma minorities in Albania is based on opportunistic (convenient) samples, and little is known about the prevalence of and the main reasons for self-perceived barriers to accessing PHC services in nationwide population-representative samples. Additionally, little is known about the number of barriers to accessing PHC services faced by minorities compared to their ethnic Albanian counterparts. Hence, despite the evidence from other European contexts highlighting disparities in healthcare access among Roma populations, there is a critical lack of context-specific data from Albania and other countries in the Western Balkans.

In this framework, given Albania's distinct sociopolitical and health system characteristics, we aimed to address this gap by examining ethnic differences in the number and nature of self-perceived barriers to accessing PHC services. We hypothesised a higher number of self-perceived barriers to accessing PHC services among Roma and Egyptian minorities compared to ethnic Albanians. Additionally, we hypothesised a higher prevalence of structural and/or communication-related barriers perceived by Roma/Egyptian minorities compared to ethnic Albanians, considering their lower socioeconomic level. The novelty of this study lies in its examination of ethnic differences in both the number and the underlying reasons for self-perceived barriers to accessing PHC services within a Western Balkan population, a region that remains markedly under-researched in the broader European context. Beyond its regional focus, this study contributes to expanding the understanding of how minority populations experience inequities in PHC access, thereby informing comparative research and policy debates across diverse European and global settings.

## 2 METHODS

A cross-sectional study was conducted in Albania during 1–31 October 2024.

### 2.1 Study population and sampling

The study population consisted of all participants who reported barriers to accessing PHC services (N = 533), which was pertinent to a population-based survey that included a nationwide representative sample of adults of both sexes who attended PHC services during October 2024. The sample for this survey was allocated across 18 sites (districts) in all four regions of Albania (northern, central, southern, and Tirana – the capital of Albania) using probability proportional to size (PPS). Within each selected health facility (n = 18; one per site), participants were recruited consecutively. Accordingly, the approach employed was designed to ensure nationwide representativeness of our study sample.

The minimum required sample size was calculated for several hypotheses related to sociodemographic correlates of self-perceived barriers to accessing PHC services, including gender and socioeconomic characteristics. The significance level (two-tailed) was set at 5%, and the study power at 80%. Based on conservative assumptions, the minimum required sample size was estimated at approximately 800 individuals, stratified by the four Albanian regions and weighted by their respective population sizes. We decided to target 1200 individuals to increase the study power and account for potential nonresponse. Of the initially targeted sample, 168 individuals were either too sick or refused to participate.

Hence, overall, the sample included in this survey consisted of 1032 adult users of PHC services (53% women; mean age: 47±18 years; overall response rate: 86%). Of these, 533 (≈52%) individuals reported barriers to accessing PHC services, thereby constituting the sample of the current analysis.

### 2.2 Data collection

Data collection consisted of an interviewer-administered semi-structured questionnaire, derived from validated instruments used in several previous studies conducted in Albania. Overall, six interviewers were involved in data collection, having been trained to employ standardised procedures, including consistent terminology and methodological approaches.

The questionnaire inquired about perceived barriers to accessing PHC services, as well as the sociodemographic characteristics and health profiles of study participants.

Initially, all participants included in this survey (N = 1032) were asked whether they had ever faced any barriers (obstacles) to accessing PHC services for themselves or their close family members. Potential responses were: “yes” vs. “no”.

Participants who responded positively constituted the current study population (N = 533) and were subsequently asked the following question: “What barriers do you face in accessing PHC services? (select all that apply): Distance to health facility; Cost of services; Lack of information; Poor service quality; Long waiting times; Transportation issues; Language barriers; Cultural barriers; Health reasons (too sick to go); No trust in the health system; Stigma; Don't know where to go; Other reasons (please specify in your own words)”.

Health profile included assessment of self-rated health of participants (trichotomised into: very poor/poor, average, and good/excellent), presence of any disability (“yes” vs. “no”), and presence of any chronic diseases/conditions (“yes” vs. “no”).

Sociodemographic characteristics included sex (men vs. women), age (in the analysis trichotomised into: 18–39, 40–59, and ≥ 60 years), place of residence (in the analysis dichotomised into: urban and/or periurban vs. rural areas), ethnicity (dichotomised into: ethnic Albanian vs. other ethnicities), marital status (dichotomised in the analysis into: “married” vs. “other”), educational attainment (trichotomised into: low, middle, and high), and economic situation (good, average, and bad).

### 2.3 Ethical aspects

The study was approved by the Ethical Council of the University of Medicine, Tirana (no. 2507/1, date: 27. 09. 2024). All participants were initially informed about the aim and procedures of the study and assured of the anonymity and confidentiality of the study. In addition, all participants were informed that the findings would be presented in aggregate form, thereby precluding the identification of personal information.

### 2.4 Consent to participate

All individuals provided oral consent to participate in the study.

### 2.5 Statistical analysis

Fisher's exact test was used to assess differences in the distribution of background characteristics (sociodemographic factors and health profile) (Table 1), as well as differences in the distribution of the number and perceived reasons for barriers to accessing PHC services (Table 2) between ethnic Albanians and other ethnicities. Conversely, a general linear model and a binary logistic regression were employed to assess the association between the number of perceived barriers to accessing PHC services and ethnicity groupings (Table 3). Crude and multivariable-adjusted mean values and their respective 95% confidence intervals (95%CIs) and p-values were calculated (upper panel: results from the general linear models), along with odds ratios (ORs: “≥ 2 barriers” vs. “one barrier only” to accessing PHC services) and their respective 95%CIs and p-values (lower panel: results from binary logistic regression). For the binary logistic regression models, we decided to dichotomise the number of barriers into “only one” vs. “≥ 2”, considering the distribution of the number of reported barriers (62 ethnic Albanians reported three or more barriers compared with only 24 ethnic minority participants; Table 2 – lower panel). All multivariable-adjusted logistic regression models met the Hosmer-Lemeshow criterion (17).

**Table 1.** Distribution of background characteristics in a sample of Albanian adults who reported barriers to accessing primary health care services in 2024 (N = 533).

**Variable**	**Total (N = 533)**	**Ethnicity**		
		**Albanian (N = 444)**	**Other (N = 89)^a^**	**P^c^**
**Sex:**				
Men	215 (40.3)^b^	159 (35.8)	56 (62.9)	< 0.001
Women	318 (59.7)	285 (64.2)	33 (37.1)	
**Age-group:**				
18–39 years	228 (42.8)	203 (45.7)	25 (28.1)	0.003
40–59 years	168 (31.5)	128 (28.8)	40 (44.9)	
≥ 60 years	137 (25.7)	113 (25.5)	24 (27.0)	
**Place of residence:**				
Urban/periurban areas	463 (86.9)	374 (84.2)	89 (100.0)	< 0.001
Rural areas	70 (13.1)	70 (15.8)	0 (–)	
**Marital status:**				
Married	334 (62.7)	264 (59.5)	70 (78.7)	< 0.001
Other	199 (37.3)	180 (40.5)	19 (21.3)	
**Education:**				
Low	161 (30.2)	85 (19.1)	76 (85.4)	< 0.001
Middle	180 (33.8)	171 (38.5)	9 (10.1)	
High	192 (36.0)	188 (42.3)	4 (4.5)	
**Economic situation:**				
Good	39 (7.3)	38 (8.6)	1 (1.1)	< 0.001
Average	324 (60.8)	296 (66.7)	28 (31.5)	
Bad	170 (31.9)	110 (24.8)	60 (67.4)	
**Self-perceived health:**				
Very poor/poor	59 (11.1)	36 (8.1)	23 (25.8)	< 0.001
Average	159 (29.8)	139 (31.3)	20 (22.5)	
Good/excellent	315 (59.1)	269 (60.6)	46 (51.7)	
**Disability:**				
No	391 (73.4)	335 (75.5)	56 (62.9)	0.018
Yes	142 (26.6)	109 (24.5)	33 (37.1)	
**Chronic conditions:**				
No	348 (65.3)	305 (68.7)	43 (48.3)	< 0.001
Yes	185 (34.7)	139 (31.3)	46 (51.7)	

Legend:

a Other ethnicities included the following: Roma (n = 57), Egyptian (n = 30), and Gorani or Macedonian (n = 2).

b Absolute numbers and their respective column percentages (in parentheses).

c P-values from Fisher's exact test.

**Table 2.** Distribution of number and perceived reasons for barriers to accessing primary health care services among study participants, by ethnicity (N = 533).

**Upper panel: distribution of *reasons* for perceived barriers**			
**Reason (type of barrier)**	**Ethnicity**		
	**Albanian (N = 444)**	**Other ethnicities (N = 89)^a^**	**P^c^**
**Cost**	121 (27.3)^b^	20 (22.5)	0.430
**Long waiting time**	105 (23.6)	15 (16.9)	0.210
**Distance**	81 (18.2)	23 (25.8)	0.108
**Poor service quality**	75 (16.9)	14 (15.7)	0.877
**Lack of information**	68 (15.3)	13 (14.6)	0.998
**Transportation**	55 (12.4)	5 (5.6)	0.068
**Lack of trust**	44 (9.9)	1 (1.1)	0.003
**Cultural barriers**	4 (0.9)	30 (33.7)	< 0.001
**Health reasons**	24 (5.4)	5 (5.6)	0.998
**Language barriers**	5 (1.1)	23 (25.8)	< 0.001
**Don't know where to go**	13 (2.9)	1 (1.1)	0.483
**Stigma**	8 (1.8)	1 (1.1)	0.999
**Other barriers^d^**	102 (23.0)	6 (6.7)	< 0.001
**Unspecified barriers**	10 (2.3)	7 (7.9)	0.013

**Lower panel: distribution of the *number* of perceived barriers**			
**Number of barriers**	**Ethnicity**		
	**Albanian (N = 444)**	**Other ethnicities (N = 89)** ^a^	**P**
**Mean (±SD) number of barriers**	1.6±1.0	1.8±0.9	0.004^e^
**Number of barriers:**			
1 barrier	271 (61.0)^f^	41 (46.1)	
2 barriers	111 (25.0)	24 (27.0)	0.002^c^
3 barriers	43 (9.7)	21 (23.6)	
≥ 4 barriers	19 (4.3)	3 (3.4)	
**Number of barriers:**			
1 barrier	271 (61.0)	41 (46.1)	0.010^c^
≥ 2 barriers	173 (39.0)	48 (53.9)	

Legend:

a Roma (n = 57), Egyptian (n = 30), and Gorani or Macedonian (n = 2).

b Absolute numbers and their respective percentages (in parentheses).

c P-values from Fisher's exact test.

d As specified by participants, “other barriers” included higher expectations than those met by the current services offered in the public PHC services in Albania.

e P-value from Mann-Whitney test.

f Absolute numbers and their respective column percentages (in parentheses).

**Table 3.** Association of the number of perceived barriers to accessing primary health care services with ethnicity; results from general linear models and binary logistic regression.

**Upper panel: general linear models**			
**Model**	**Mean^a^**	**95%CI^a^**	**P^a^**
**Model 1^b^**			
Albanian	1.61	1.52–1.70	0.042
Other ethnicities	1.84	1.64–2.05	
**Model 2 ^c^**			
Albanian	1.62	1.53–1.72	0.074
Other ethnicities	1.83	1.63–2.04	
**Model 3 ^d^**			
Albanian	1.62	1.52–1.71	0.087
Other ethnicities	1.82	1.61–2.02	
**Model 4 ^e^**			
Albanian	1.64	1.51–1.76	0.097
Other ethnicities	1.87	1.64–2.10	
**Model 5 ^f^**			
Albanian	1.69	1.55–1.83	0.111
Other ethnicities	1.91	1.68–2.14	

**Lower panel: logistic regression models**			
**Model**	**OR^g^**	**95%CI^g^**	**P^g^**
**Model 1 ^b^**			
Albanian	1.00	reference	0.010
Other ethnicities	1.83	1.16–2.90	
**Model 2 ^c^**			
Albanian	1.00	reference	0.015
Other ethnicities	1.79	1.12–2.86	
**Model 3 ^d^**			
Albanian	1.00	reference	0.020
Other ethnicities	1.75	1.09–2.82	
**Model 4 ^e^**			
Albanian	1.00	reference	0.022
Other ethnicities	1.97	1.10–3.50	
**Model 5 ^f^**			
Albanian	1.00	reference	0.028
Other ethnicities	1.93	1.07–3.48	

Legend:

a Mean values and their respective confidence intervals (95%CIs) and p-values from the general linear models.

b Model 1: crude/unadjusted.

c Model 2: adjusted for sex (men vs. women).

d Model 3: adjusted for sex and age (18–39 years, 40–59 years, ≥ 60 years).

e Model 4: adjusted for all sociodemographic factors [sex, age, marital status (married vs. single/divorced/widowed), education (low vs. middle/high), and economic situation (bad vs. average/good)].

f Model 5: adjusted for sociodemographic factors and health profile [self-perceived health status (very poor/poor, average, good/excellent), disability (yes vs. no), and chronic conditions (yes vs. no)].

g Odds ratios (ORs: “≥ 2 barriers” vs. “one barrier only” to accessing primary health care services) and their respective 95%CIs and p-values from binary logistic regression analysis.

P ≤ 0.05 was considered as statistically significant for all statistical tests. Statistical Package for the Social Sciences (SPSS, version 19.0) was employed for all the statistical analyses.

## 3 RESULTS

Of 533 study participants, 444 (83.3%) were ethnic Albanians, whereas the remaining 89 (16.7%) individuals were of other ethnicities [Roma (n = 57), Egyptian (n = 30), and Goran or Macedonian (n = 2)]. The overall mean age of study participants was 44.9±18.3 years (44.1±18.8 years among ethnic Albanians vs. 48.5±14.9 years in other ethnicities; Mann-Whitney test: P = 0.024) [data not shown in the tables].

The distribution of background characteristics in the overall sample and specifically by ethnic groups is presented in Table 1.

Overall, about 60% of study participants were women; one-fourth was aged 60 years and above; 13% were rural residents; almost two-thirds were currently married (63%); almost one-third reported a low educational attainment (30%) and/or a bad economic situation (32%); 59% perceived their general health as good and/or excellent; around 27% reported at least one type of disability; and more than one-third (≈35%) reported a chronic condition. The proportion of women and younger individuals was substantially lower among minorities than among ethnic Albanians (both P < 0.01). None of the minority participants resided in rural areas, which is a well-known fact in Albania, as Roma and Egyptian communities tend to reside mostly in urban and periurban areas. Hence, this variable was not considered in further analyses. The proportion of married individuals was significantly higher among minorities compared to ethnic Albanians. Furthermore, the prevalence of low education, poor economic circumstances, poor health, disability, and chronic conditions was significantly higher among participants from minority groups than among ethnic Albanians (all P < 0.01).

The most common perceived barrier to accessing PHC services in both groups was the cost of services (≈27% in ethnic Albanians vs. ≈23% in minority groups) [Table 2 – upper panel], followed by long waiting time, which was also more prevalent among Albanians (≈24%) than ethnic minorities (≈17%), although findings were not statistically significant. Conversely, distance was reported as a barrier among ≈26% of ethnic minorities, compared with 18% of Albanians, a difference that was only borderline statistically significant (P = 0.11). Perceptions regarding service quality were similar across groups, as was the perceived lack of information. Transportation was reported as a greater barrier for Albanians than for ethnic minorities (≈12% vs. ≈6%, respectively; P = 0.07). Furthermore, lack of trust was significantly more prevalent among Albanians than ethnic minorities (≈10% vs. ≈1%, respectively; P < 0.01), presumably reflecting internal system scepticism. On the other hand, cultural barriers were considerably more prevalent among minority groups than among Albanians (≈34% vs. ≈1%, respectively; P < 0.01), a profound disparity that denotes the cultural mismatch as a major barrier for minorities in Albania. Likewise, language barriers were reported by ≈26% of participants from minority groups, compared with only ≈1% among ethnic Albanians (P < 0.01), indicating a critical barrier for minorities given the absence of multilingual services. In turn, the prevalence of “other” barriers (i.e., higher expectations than those currently met by the public PHC system in Albania, as specified by participants in their own words) was substantially higher among Albanians than among ethnic minorities (23% vs. ≈7%, respectively; P < 0.01).

Minority groups reported a significantly higher mean number of barriers to accessing PHC services compared to ethnic Albanians (1.8 vs. 1.6, P < 0.01) [Table 2 – lower panel]. Additionally, while most Albanians (61%) experienced only one barrier, minority respondents were more likely to face multiple barriers (≈54%). More specifically, ≥ 2 barriers were reported by ≈54% of minorities, compared with only 39% of Albanians (P = 0.01).

In general linear models (Table 3 – upper panel), the mean number of barriers to accessing PHC services was higher in minority groups compared to ethnic Albanians for all models (models 1–5), but findings were only borderline statistically significant [fully adjusted model (model 5): 1.9 vs. 1.7, respectively; P = 0.11].

In binary logistic regression models (Table 3 – lower panel), the odds of reporting two or more barriers to accessing PHC services were 83% higher (P = 0.01) among minority groups than in ethnic Albanians (model 1).

Adjustment for sex and next for age of participants (models 2–3) slightly attenuated the estimates. Conversely, further adjustment for the other sociodemographic factors (model 4) accentuated the findings (OR ≈ 2.0, P = 0.02). Upon additional adjustment for health characteristics of study participants (model 5), the odds of reporting two or more barriers to accessing PHC services were 93% higher (OR = 1.9, 95%CI = 1.1–3.5) among minority groups than in ethnic Albanians.

## 4 DISCUSSION

### 4.1 Main findings

Our study involved a nationwide representative sample of PHC users who reported barriers to accessing PHC services. Minority groups exhibited greater social vulnerability, with markedly higher levels of low educational attainment, economic hardship, poor health status, disability, and chronic conditions. Overall, the cost of services, a key structural barrier, was the most commonly perceived obstacle across all ethnic groups. Ethnic Albanians reported more frequently a lack of trust in the system and dissatisfaction related to expectations, indicating greater systemic barriers in this group. In contrast, minority participants (almost entirely pertinent to Roma and Egyptian communities) reported significantly higher levels of cultural and language barriers, underscoring communication-related obstacles and the absence of multilingual services in transitional Albania. Furthermore, minority groups faced a greater cumulative burden of access barriers, with a higher proportion reporting multiple challenges. Regression analyses revealed that minorities had significantly increased odds of encountering two or more barriers, even after adjusting for a range of sociodemographic and health-related factors. Nevertheless, given the cross-sectional nature of the study, the associations identified between ethnicity and reported barriers cannot establish causality but only highlight correlations that may be influenced by unmeasured contextual factors.

### 4.2 Comparison with previous studies

In our study, the cost of health services was the most prevalent barrier to accessing PHC services in ethnic Albanians and Roma/Egyptian minorities. The latter is in line with a previous report from countries of Central and Eastern Europe, including Albania, where the main reason for unmet health needs among Roma minorities consisted of affordability of care (4). Hence, the cost of services is the main reason for the remarkably high prevalence of unmet needs among Roma minorities (4). In Albania, Roma individuals have been reported more than twice as likely to experience unmet healthcare needs compared to their non-Roma counterparts, mainly due to service cost (4, 18). Likewise, mainly due to affordability issues, the odds of experiencing unmet healthcare needs have been reported significantly higher among Roma minorities compared with non-Roma groups in: Bosnia and Herzegovina (4, 18); Bulgaria (4, 18, 19); Montenegro (4, 18); North Macedonia (4, 18); Romania (4, 18, 20, 21); and Serbia (4, 18).

In principle, PHC users in Albania are not required to pay for services. However, medications prescribed by family physicians that fall within the reimbursement list are often perceived as ineffective, prompting patients to incur substantial out-of-pocket expenses for alternative medicines not covered by reimbursement. In addition, informal payments may represent another significant financial barrier to accessing PHC services in Albania.

Our findings related to a higher prevalence of communication-related barriers to accessing PHC services, including language and cultural barriers among Roma/Egyptian minorities, are generally compatible with previous reports from Albania (15, 16). Conversely, our study did not reveal a significant difference between stigma and minority status, as documented in previous studies conducted in Albania (15, 16). The lack of relationship in our study may reflect underlying collinearity, in which stigma is indirectly embedded within more salient, structurally mediated dimensions of exclusion, such as language and cultural barriers.

Our findings related to Roma/Egyptian minorities also resonate with other marginalised groups, such as migrants, refugees, and other population groups in fragile settings, which remain underserved and face significant barriers to accessing healthcare services (12, 22). In particular, language barriers are a major obstacle to migrant healthcare access, and therefore translation and cultural mediation are essential to overcoming these barriers and ensuring equitable care (12).

On the other hand, in our study, ethnic Albanians perceived more systemic barriers than Roma/Egyptian minorities, including a lack of trust in the public system and expectations-related dissatisfaction. These perceived barriers may reflect greater system awareness among ethnic Albanians, who, unlike minorities, may be more accustomed to navigating public services and thus more likely to appraise systemic weaknesses critically. Also, it may reflect greater reliance on private healthcare services among ethnic Albanians, whose higher expectations may predispose them to perceive the public sector more negatively and criticise its shortcomings, as suggested by previous studies in Albania (23, 24).

Our findings related to adult Roma/Egyptian users of PHC services are also compatible with recent evidence from Albania pertaining to children aged 12–15 years (25, 26). In this age-group, Roma/Egyptian children reported: a significantly higher prevalence of barriers to accessing healthcare services (51% vs. 42% among ethnic Albanians), with distance and non-availability of the parents as main barriers (25); and a higher prevalence of poor attitudes toward health promotion (ability to maintain and improve health: 33% vs. 29%, respectively) (26). Furthermore, among Albanian children aged 11–15 years, the prevalence of unhealthy dietary habits (including breakfast skipping and low consumption of fruits and vegetables) has been reported to be lower among lower socioeconomic groups, a population segment that includes Roma/Egyptian minorities (27).

### 4.3 Policy implications

In our study, ethnic minorities reported markedly higher barriers related to culture and language, indicating communication-related obstacles which reflect deeper inequities in the organisation of health services in post-communist Albania. Hence, there is an urgent need for culturally sensitive and language-accessible services in Albania (15). From this perspective, targeted, culturally sensitive, linguistically accessible, and community-based educational programs are needed to increase health literacy levels and system navigation skills among ethnic minorities in Albania and other European countries (15). Furthermore, to address the documented access inequities, policy responses in Albania should prioritise the development of multilingual PHC staff training programs, the systematic inclusion of Roma mediators in community health outreach, and the incorporation of equity monitoring indicators into PHC performance evaluation frameworks. In turn, ethnic Albanian participants were seemingly more affected by systemic barriers, suggesting the need for reforms in service delivery. In particular, the significantly higher expectations than those met by the current public PHC services among ethnic Albanians highlight the need for policy measures that enhance the responsiveness and quality of public PHC services, particularly by aligning service delivery with the expectations of more system-engaged populations such as ethnic Albanians, while ensuring equity across all groups (28, 29).

Conversely, our finding that the cost of health services was the most commonly perceived structural barrier to accessing PHC services across all ethnic groups highlights the urgent need for policy interventions that reduce financial barriers to PHC (such as expanding coverage, subsidising essential services, or introducing targeted fee exemptions) to ensure equitable access across all ethnic groups (30).

Overall, our findings reflect broad challenges in the Albanian health system's responsiveness and the persistent inequities shaping access to PHC in a transitional post-communist health system. The concentration of structural, cultural, and communicative barriers among Roma and Egyptian minorities illustrates a pattern of differential responsiveness, in which services fail to adequately adapt to the social, linguistic, and cultural needs of marginalised communities, thereby undermining equitable access (31). Conversely, the prominence of cost-related barriers across all ethnic groups points to inadequate financial protection in Albania, which is an essential component of a responsive and equitable health system (30, 31). In turn, the greater distrust and expectations-related dissatisfaction reported by ethnic Albanians highlight how unmet expectations also signal weaknesses in responsiveness at the interface of service quality, provider-patient interactions, and system accountability (30, 31). Together, these patterns suggest that barriers to PHC in Albania are not merely individual experiences but manifestations of systemic asymmetries in how the health system accommodates diverse populations, reinforcing the need for reforms that strengthen equity, cultural competence, and the capacity of services to respond appropriately to PHC users' expectations and needs (30, 31).

### 4.4 Study limitations

The cross-sectional design restricts causal inference; while associations between ethnicity and perceived barriers were identified, temporal relationships cannot be established. Additionally, the data collection process was restricted to October 2024. Extending the study duration to three months could have increased participation and improved the representativeness of the sample. Also, reliance on self-reported data may introduce recall and social desirability biases, particularly in sensitive domains such as trust and stigma. In particular, social desirability bias may have led minority participants to underreport barriers they perceive as socially undesirable (e.g., trust, stigma, or discrimination), potentially attenuating the observed ethnic differences in these important barriers. Conversely, recall bias may have led participants to overemphasise more memorable difficulties (e.g., cost, distance, or transportation), potentially exaggerating the share of these structural barriers across all ethnic groups. On another matter, because participants were asked not only about their own experiences but also about those of close family members, responses may have been subject to recall bias or misrepresentation, as individuals might inaccurately perceive or report barriers others face. Additionally, although the sampling strategy ensured national representation, the relatively small sample size (n = 553) may restrict the generalizability of our findings across diverse population groups. Especially, the small number of minority participants (n = 89), particularly non-Roma and non-Egyptian groups (only two participants), limits subgroup analysis and may obscure intra-group heterogeneity. Overall, the relatively small sample size and limited representation of minority groups may have influenced our results by underrepresenting key subpopulations and reducing statistical power; therefore, our findings should be interpreted as indicative rather than definitive, highlighting trends that warrant further investigation in larger, more balanced samples. Furthermore, the questionnaire did not capture the frequency or severity of barriers, nor did it explore institutional or provider-level factors that may contribute to deeper inequities. Lastly, the absence of qualitative data restricts a deeper understanding of cultural and linguistic mismatches, which were shown to affect minority groups in Albania disproportionately. Hence, there is a need for longitudinal, mixed-methods research to confirm and, eventually, expand the findings of our study.

## 5 CONCLUSIONS

Roma and Egyptian minorities in transitional Albania face significantly greater self-perceived barriers to accessing PHC services compared to ethnic Albanians. While structural barriers are similarly reported across groups, communication-related barriers disproportionately affect minorities, whereas Albanians more commonly perceive systemic shortcomings. Our findings underscore the need for targeted equity-focused interventions to address persistent disparities in health care access in Albania and in similar transitional settings. Our findings resonate with the WHO “leave no one behind” agenda (22) and align with the EU Roma inclusion strategies (32), highlighting the imperative of embedding equity and minority-sensitive measures into health system reforms.
